# Potential Pathogenicity of *Aeromonas* spp. Recovered in River Water, Soil, and Vegetation from a Natural Recreational Area

**DOI:** 10.3390/pathogens11111382

**Published:** 2022-11-19

**Authors:** Roberto M. Guerra, Francisco Damián Maleno, Maria José Figueras, Isabel Pujol-Bajador, Ana Fernández-Bravo

**Affiliations:** 1 Unit of Microbiology, Department of Basic Health Sciences, Faculty of Medicine and Health Sciences, IISPV, University Rovira i Virgili, 43201 Reus, Spain; 2 Microbiology Laboratory, University Hospital Sant Joan de Reus, Salut Sant Joan de Reus-Baix Camp, 43204 Reus, Spain

**Keywords:** *Aeromonas*, river water, pathogenicity, macrophages

## Abstract

The genus *Aeromonas* is widely distributed in aquatic environments and is recognized as a potential human pathogen. Some *Aeromonas* species are able to cause a wide spectrum of diseases, mainly gastroenteritis, skin and soft-tissue infections, bacteremia, and sepsis. Currently, untreated river water is used for irrigation and recreational purposes. In this study, the *Aeromonas* spp. present in a river recreational environment was investigated by quantifying its presence in water, soil, and vegetation using three techniques: qPCR, plate counting in selective ADA medium, and Most Probable Number, in parallel. The presence of clones in the three types of samples was elucidated through genotyping with the ERIC-PCR technique, whereas the identification of the isolated *Aeromonas* was carried out by sequencing the *rpoD* gene. Finally, the pathogenic potential of some of the strains was explored by studying the presence and expression of virulence genes characteristic of the genus, their antimicrobial susceptibility profile, as well as the quantification of their cell damage and intracellular survival in an in vitro macrophages infection model. The results showed the presence of *Aeromonas* in all samples with the three quantification methods, with *Aeromonas popoffii* being the most prevalent species. The presence of strains with the same genotype (ERIC-PCR) was also confirmed in different samples. Some of the strains showed a high level of cell damage and intracellular bacterial survival, as well as the presence of various virulence factors. Furthermore, these strains showed resistance to some of the antibiotics tested and used therapeutically in both humans and animals. These results indicate that the presence of *Aeromonas* in this environment may represent a biosanitary risk that could be a public health problem.

## 1. Introduction

The genus *Aeromonas* comprises a group of Gram-negative bacteria autochthonous to aquatic environments and widely distributed in numerous ecosystems, including groundwater, drinking water, bottled water, river water, seawater, irrigation water, and reclaimed wastewater [1,2,3,4,5,6,7,8,9,10]. However, these bacteria have also been recovered from soil, animals, food products, and humans infections in immunocompromised and immunocompetent patients [11,12]. Cases of severe *Aeromonas* infections have been reported, mainly due to contact with contaminated waters in rivers and lakes [13,14,15,16,17,18]. The most frequent *Aeromonas* infections are gastroenteritis and skin and soft-tissue infections, followed by bacteremia and sepsis, as well as other infections that affect the hepatobiliary system, respiratory tract, bones, and joints [12,19].

Water is a limited resource, so currently waters from different sources, such as reclaimed water or untreated river water, are used for irrigation [8,10]. Previous literature has demonstrated the presence of the same *Aeromonas* strains (clones) in vegetables and in the irrigation water used, as well as in areas close to these water sources [8], which may represent a health problem [8,20,21]. In addition, a very severe case of *Aeromonas* necrotizing fasciitis in a young healthy girl was reported after she fell into a river [17,18]. The progression of the infection led to the amputation of a large part of her four limbs.

The pathogenesis of *Aeromonas* infections is complex and considered multifactorial. Several genes encoding for virulence factors link to the capacity of *Aeromonas* to evade the host immune response and contribute to the infectious process [19,22]. These include cell structural components; extracellular proteins like aerolysins, hemolysins, lipases, cytolytic and cytotonic enterotoxins; secretion systems; and metal-associated proteins [22,23]. Another concerning characteristic of *Aeromonas* is its increasing resistance to antibiotics, resulting in treatment failure in human and animal infections [11,24]. A wide diversity of genetic elements responsible for antimicrobial resistance in *Aeromonas* has been described, which can be encoded in the chromosome, in mobile genetic elements, or integrons [25,26,27,28].

In the natural area “El Clot de la Mare de Déu” (Burriana, Spain), which belongs to the fluvial area of the Mijares river, untreated and uncontrolled waters are frequently used for agricultural irrigation and for recreational purposes. The present study quantifies the presence and diversity of *Aeromonas* species in the river, soil, and vegetation of the recreational area and characterizes the presence and expression of virulence genes, the antimicrobial susceptibility profile, as well as the survival and infective capacity of the *Aeromonas* strains in macrophages, in order to evaluate the potential risk to human health associated with the use of this water environment.

## 2. Material and Methods

### 2.1. Sample Collection and Processing

Three types of samples were analyzed from “El Clot de la Mare de Déu” (Burriana, Spain): water, soil, and vegetation. The water sample was collected in a 2 L polypropylene bottle, while the soil and vegetation samples were collected in polyethylene bags. All of them were refrigerated and transported to the laboratory and processed on the same day as collection. The water sample was serial diluted, while the soil and vegetation samples were mixed with distilled water, vortexed, and a serial dilution was performed. 

### 2.2. Aeromonas Quantification by Plate Counting

Aliquots of 100 µL of each sample were plated on the surface of Ampicillin Dextrin Agar (ADA, HiMedia, Mumbai, India). After incubation of the plates at 37 °C for 24 h, the colony-forming units (CFU) were counted [8,12].

### 2.3. Aeromonas Quantification by qPCR

DNA was extracted from all samples using the Easy-DNA^TM^ kit (Invitrogen, Carlsbad, CA, USA). Quantitative real-time PCR was performed with purified DNA using the DNA TargetSpecies dtec-qPCR kit for *Aeromonas* species (Genetic PCR solutions, Orihuela, Spain) and the SteponePlus^TM^ Real-Time PCR system (Applied Biosystems, Waltham, MA, USA). The number of copies was calculated based on the standard line and the corresponding amplification cycle threshold (Ct). 

### 2.4. Aeromonas Quantification by Most Probable Number (MPN)

All samples were quantified via a 3-tube MPN method, inoculating 500 μL of the serial dilutions by triplicate in tubes with 2.5 mL of Alkaline Peptone Water supplemented with ampicillin at a concentration of 10 mg/L. After incubating the tubes for 24 h at 37 °C, the number of positives (tubes that presented yellow/orange turbidity) was counted and the most probable number was obtained with the “Most Probable Number Calculator” computer tool (https://mostprobablenumbercalculator.epa.gov/mpnForm, accessed on 1 March 2021). Later, the tubes were plated in ADA as a control to demonstrate the presence of *Aeromonas* [8,12]. 

### 2.5. Bacterial Strains Maintenance and Culture Conditions

From the ADA plates, 24 colonies were selected based on the typical morphology of *Aeromonas,* i.e., yellow colonies grown on this medium. Bacteria were subcultured on Difco^TM^ Tryptic Soy Agar (TSA; Becton Dickinson and Company, Sparks, MD, USA), performing successive passages to obtain pure cultures. For conservation, the strains were maintained in Difco^TM^ Tryptic Soy Broth (TSB; Becton Dickinson and Company) plus glycerol (15%) at −80 °C. Before experiments, bacteria were routinely grown in TSA at 37 °C for 24 h. 

### 2.6. DNA Extraction and Genus-Level Identification Based on GCAT Gene

The genomic DNA of the bacterial strains was extracted from pure cultures grown in TSA using the InstaGene^TM^ DNA purification matrix (Bio-Rad, Hercules, CA, USA) and following the manufacturer’s instructions. The 24 strains were identified as *Aeromonas* or not via the detection of the GCAT gene, which encodes a glycerophospholipid cholesterol acyl transferase specific to this genus, using the primers and conditions described by Chacón et al. [29] (Table 1). The PCR products with the expected amplicons size of 237 bp [29] were verified in a 1% agarose gel electrophoresis. Gels were stained using RedSafe^TM^ nucleic acid staining solution (iNtRON biotechnology, Seongnam, Korea) and visualized using a transilluminator (Molecular Imager^®^ Gel Doc^TM^ XRT) and the Image Lab^TM^ Software, both from Bio-Rad.

### 2.7. Genotyping of Aeromonas Strains

All the *Aeromonas* strains were genotyped using the Enterobacterial Repetitive Intergenic Consensus Polymerase Chain Reaction (ERIC-PCR) technique, using the primers described by Versalovic et al. [30] (Table 1) and conditions described by Houf et al. [37]. Amplicons of different sizes generated during amplification of genomic DNA were separated using 2% agarose gels, and both gel staining and band visualization were performed in the same manner as described above. Patterns with at least one band difference were considered as different genotypes [8].

### 2.8. Identification of the Aeromonas Species Based on the rpoD Gene

All the *Aeromonas* strains positive for the GCAT gene were identified by sequencing the *rpoD* gene, using the primers and conditions described by Soler et al. [31] (Table 1). The PCR products were sequenced and subsequently aligned with the *rpoD* sequences of the type strains of 36 described *Aeromonas* species using the ClustalW algorithm [38] in MEGA v6.0 [39]. The phylogenetic analysis was performed with the neighbor-joining (NJ) algorithm in MEGA v6.0.

### 2.9. Detection of Virulence Genes

For the subsequent experiments, six strains (A6 and A7, isolated from water; T8 and T10, isolated from soil; and MV8 and MV11, isolated from vegetation) were selected. The presence of five virulence genes (*lafA*, *alt*, *ast*, *stx1*, and *ascF-G*) was studied in the six selected strains by PCR using the primers (Table 1) and conditions described by Lee et al. [34].

### 2.10. Macrophages Growth Conditions and Infection

The cell line J744A.1 from mouse BALB/C monocyte/macrophage was used for the infection experiments [40]. The cells were maintained in adhesion in Dulbecco’s Modified Eagle’s Medium (DMEM; PAA Laboratories GmbH, Munich, Germany) (pH = 8) supplemented with 10% fetal bovine serum (FBS; PAA Laboratories GmbH) plus 1% P/S solution (penicillin-streptomycin stock; PAA Laboratories GmbH) at 37 °C and 5% CO_2_. Prior to infection, cells were seeded in tissue culture plates (1 × 10^6^ cells/mL) containing serum and antibiotic-free DMEM (serum-starvation conditions) for 24 h to form confluent monolayers [41]. Then, macrophages were infected with the six *Aeromonas* strains, from overnight cultures, at MOI (multiplicity of infection) 10. Two strains from a previous study, *Aeromonas veronii* 123384 and *Aeomonas jandaei* AE214, were used as controls [42]. 

### 2.11. Analysis of the Expression of ascF-G and ast Genes after Infecting Macrophages

The expression of two different genes implicated in the virulence, *ascF-G* (associated with the Type III secretion system) and *ast* (cytotonic enterotoxin), was studied after infection of the macrophage cell line J744A.1 with each *Aeromonas* strain. The primers used to evaluate the expression of the selected genes are shown in Table 1. After 3 h of infection at MOI 10, total RNA was isolated from *Aeromonas* cultures using TRIzol^®^ Reagent (Invitrogen) as previously described [43]. RNA quality and integrity were confirmed using NanoDrop 2000. The cDNA was transcribed from RNA using iScript cDNA Synthesis Kit (Bio-Rad) following the manufacturer’s instructions. Quantitative Real-Time PCR was performed in triplicate using Real-Power SYBR^®^ green PCR Mastermix (Applied Biosystems) on a StepOnePlus™ Real-Time PCR System (Applied Biosystems). Threshold cycle (CT) values were obtained to establish the relative RNA levels of the tested genes, using the 16S rRNA gene as a housekeeping gene, and then calculated with the 2^−ΔΔCt^ method.

### 2.12. Quantification of Cell Damage in Macrophages

Co-cultures of bacteria-macrophages were incubated at 37 °C and 5% CO_2_ for 1 h. Then, the medium was removed and fresh medium was added to the wells and incubated at 37 °C and 5% CO_2_ for 5 h. To determine cell damage, the amount of lactate dehydrogenase (LDH) enzyme in the supernatant was quantified using the commercial CytoTox 96^®^ Non-Radioactive Cytotoxicity Assay Kit (Promega, Madison, WI, USA), following the protocol specified by the manufacturer. Recombinant bovine LDH was used to generate a standard curve and sample values were extrapolated from there [42].

### 2.13. Intracellular Bacterial Survival of Aeromonas Strains in Macrophages

The quantitative determination of bacteria present within the macrophages was determined by the gentamicin exclusion assay [44]. Briefly, co-cultures of bacteria-macrophages were incubated at 37 °C and 5% CO_2_ for 1 h. Then, gentamicin (50 μg/mL) was added to wells for killing extracellular bacteria. After 45 min, the medium was removed, and fresh medium was added to the wells and incubated at 37 °C and 5% CO_2_ for 3 h. To determine the number of bacteria, serial dilution, followed by culturing on TSA plates, was carried out. The percentage of intracellular bacterial survival was calculated with the number of colony-forming units at time 0 h in relation to the initial dose of infection [42].

### 2.14. Antimicrobial Susceptibility Profile

The in vitro antimicrobial susceptibility profile of the six *Aeromonas* strains against five antibiotics (ampicillin, cefuroxime, ceftriaxone, tetracycline, and piperacillin/tazobactam) was determined by the Kirby–Bauer disk diffusion method according to the procedure described by Clinical and Laboratory Standards Institute [45], using antibiotic BD BBL™ Sensi-Disc™ disks (Becton Dickinson and Company), Difco^TM^ Mueller–Hinton agar medium plates (Becton Dickinson and Company), and incubation at 37 °C for 16–18 h. CLSI breakpoints were used to categorize an isolate as susceptible, intermediate, or antibiotic resistant.

### 2.15. Statistical Analysis

All experiments were performed in triplicate and significant differences were determined using Student’s two-tailed t-test and two-way ANOVA calculated on Graph Pad Prism 6.0 (GraphPad Prism Software Inc., San Diego, CA, USA). *p*-values < 0.05 were considered statistically significant (*).

## 3. Results

### 3.1. Aeromonas Quantification

**Figure 1:**
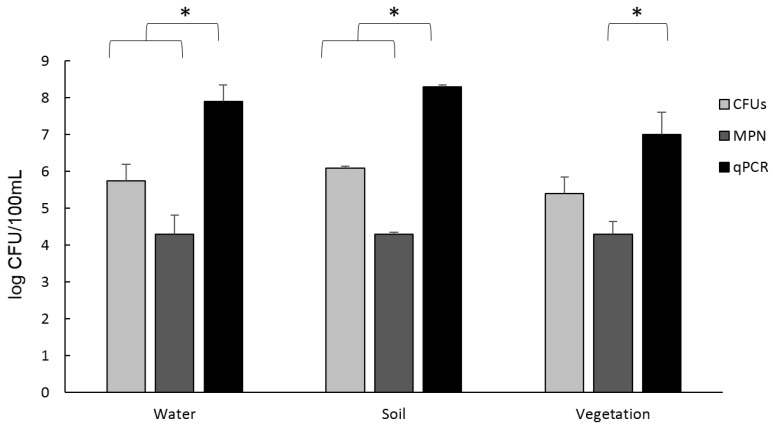
*Aeromonas* quantification by Plate Counting in ADA medium (CFUs), qPCR, and MPN for the 3 types of samples. Results are means ± SD from three independent experiments. * Significant differences *p* < 0.05.

The results of the quantification of the presence of *Aeromonas* in the samples are shown in Figure 1. Although the soil samples showed a greater number of *Aeromonas* compared to the other samples, no significant differences were observed between samples. The technique that quantified a greater quantity of *Aeromonas* was qPCR, with an average of the three samples of 9.15 × 10^7^ CFU/100 mL, followed by the Plate counting in ADA medium with 7.11 × 10^5^ CFU/100 mL and the MPN of 2.2 × 10^4^ CFU/100 mL. In the case of water and soil samples, a significant difference was shown between the qPCR and the Plate Counting method and the MPN (*p* < 0.05). In vegetation samples, only significant differences were observed between MPN and qPCR (*p* < 0.05).

### 3.2. Genus-Level Identification Based on the GCAT Gene

The presence of the GCAT gene was detected in 19 of the 24 (79%) selected isolates grown in ADA medium. Of the 19 presumptive *Aeromonas* isolates nine were from water (A1, A2, A4, A5, A6, A7, A9, A14, A18), seven from soil (T3, T4, T5, T6, T8, T9, T10), and three from vegetation (MV8, MV11, and MV18).

### 3.3. Genotyping of Aeromonas Isolates

**Figure 2:**
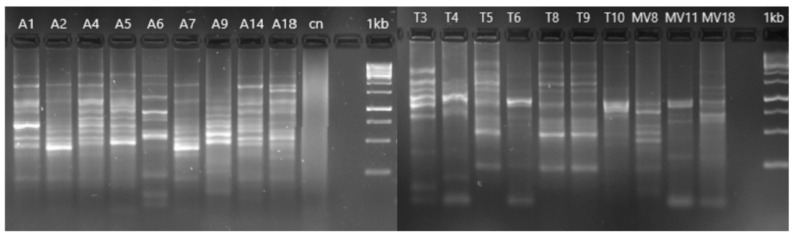
ERIC-PCR profile of the 19 *Aeromonas* strains isolated in the present study. The ERIC-PCR analysis was used to determine the genetic similarity of isolates from water (A1, A2, A4, A5, A6, A7, A9, A14, and A18), soil (T3, T4, T5, T6, T8, T9, and T10) and vegetation (MV8, MV11 and MV18). Identical band patterns between isolates indicated a clonal relation.

The ERIC-PCR analysis showed some clonal relations of the different isolates (Figure 2). These relations corresponded to isolates of water (isolated A1 and A2; A5 and A14), soil (T5, T8 and T9; T6 and T10), and vegetation (MV11 and MV18). In addition, some of the water and soil isolates were found to be identical, e.g., A6 and T3, as well as isolates A1 and A2 and T5, T8, and T9 soil isolates. 

### 3.4. Species-Level Identification Based on the rpoD Gene

**Figure 3:**
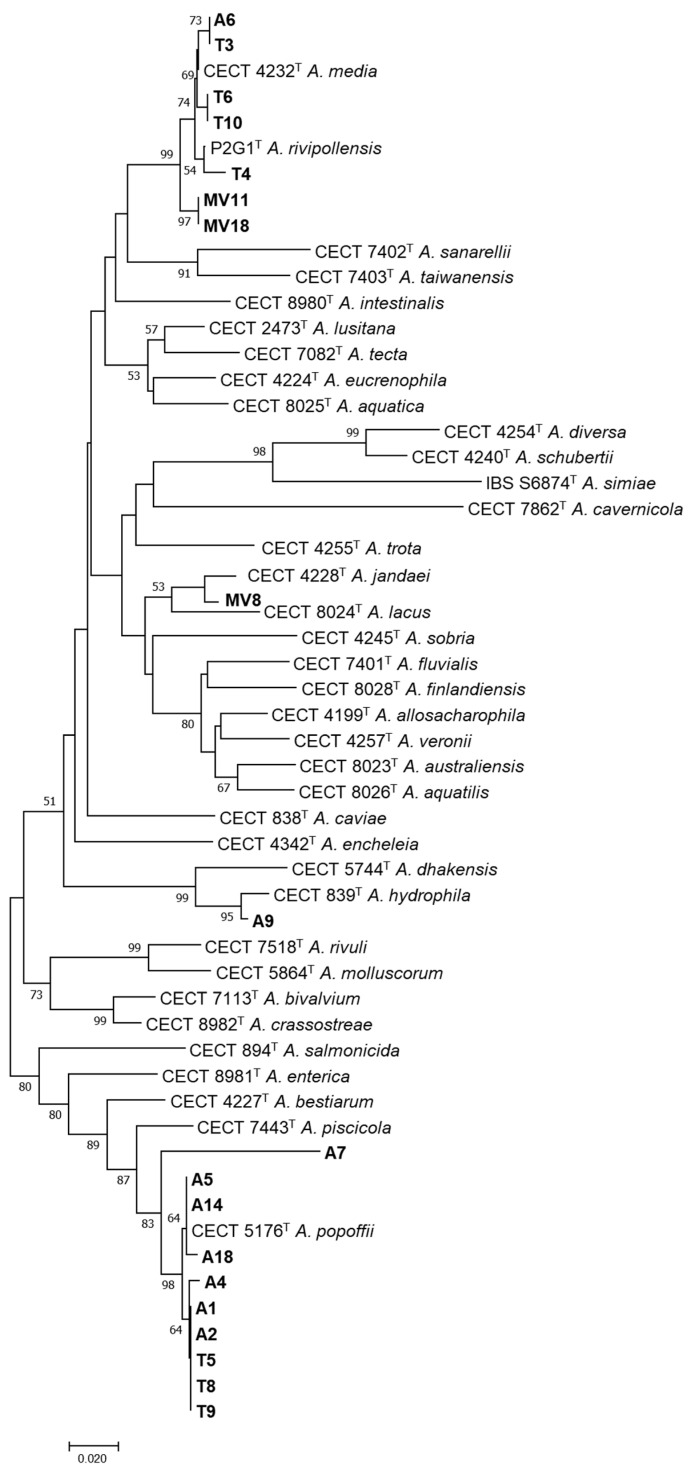
Neighbor-joining phylogenetic tree constructed with the *rpoD* gene sequences of the type strains of all known *Aeromonas* species and with the sequences of the 19 isolates recovered from water (A1, A2, A4, A5, A6, A7, A9, A14, and A18), soil (T3, T4, T5, T6, T8, T9, and T10) and vegetation (MV8, MV11 and MV18). Bootstrap percentages of more than 50% based on 1000 replications are shown at branch nodes. Bar, 0.02 substitutions per nucleotide position.

Phylogenetic analysis of the *rpoD* gene confirmed that the 19 strains belonged to the genus *Aeromonas* (Figure 3). The results showed that nine of the strains were identified as *A. popoffii* (T5, T8, T9, A1, A2, A4, A5, A14, A18), one strain as *A. hydrophila* (A9), four strains as *A. media* (A6, T3, T6, T10), one as *A. rivipollensis* (T4), and one as *A. jandaei* (MV8). Two strains, MV11 and MV18, were included in the clade of *A. media* and *A. rivipollensis* and clustered with *Aeromonas* sp. genomospecies *paramedia*. The A7 strain was identified as *Aeromonas* sp., forming a different clade from its closest species. These results were in agreement with the genotyping results, describing some of the isolates as clones.

### 3.5. Identification of Virulence Genes 

The presence/absence of the five virulence genes studied in the selected six strains (A6 and A7 from water; T8 and T10 from soil; and MV8 and MV11 from vegetation) is shown in Table 2. The results showed that the six strains (100%) possess the *laf* gene that encodes for the lateral flagellum, while the other genes were not present in all strains. Cytotonic enterotoxins *alt* and *ast* were found in four (66.6%) and five (83.3%) of the strains, respectively. The *ascF-G* gene, associated with the Type III secretion system, was present in four of the strains (66.6%). Finally, the *stx1* gene, encoding for Shiga toxin type 1 was not found in any of the strains.

### 3.6. Virulence-Associated Gene Expression

**Figure 4:**
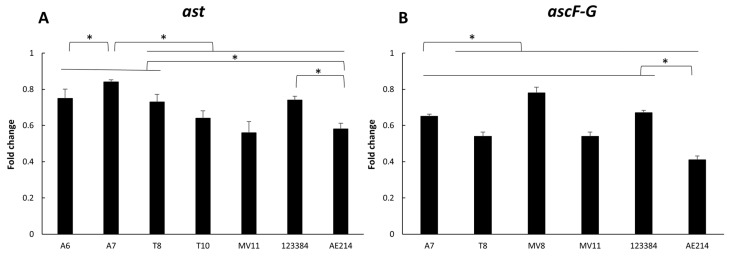
Gene expression of *ast* **(A)** and *ascF-G* **(B)** genes in the six selected *Aeromonas* strains (A6 and A7 from water; T8 and T10 from soil; and MV8 and MV11 from vegetation) and in *Aeromonas veronii* 123384 and *Aeromonas jandaei* AE214, after macrophage J744A.1 infection. Results are expressed as the mean of qPCR values. * Significant differences compared with non-infected cells *p* < 0.05.

Expression of *ast* and *ascF-G* genes is shown in Figure 4. The results showed a higher expression of both genes in the strains recovered from water and vegetation A7 (*Aeromonas* sp.) and MV8 (*A. jandaei*), respectively, showing significant differences with the other strains and the control strain *A. veronii* 123384, after 3 h of infection (*p* < 0.05). In addition, all strains studied showed a higher expression of both genes in comparison with the other control strain, *A. jandaei* AE214 (*p* < 0.05).

### 3.7. Quantification of Cell Damage in Macrophages

**Figure 5:**
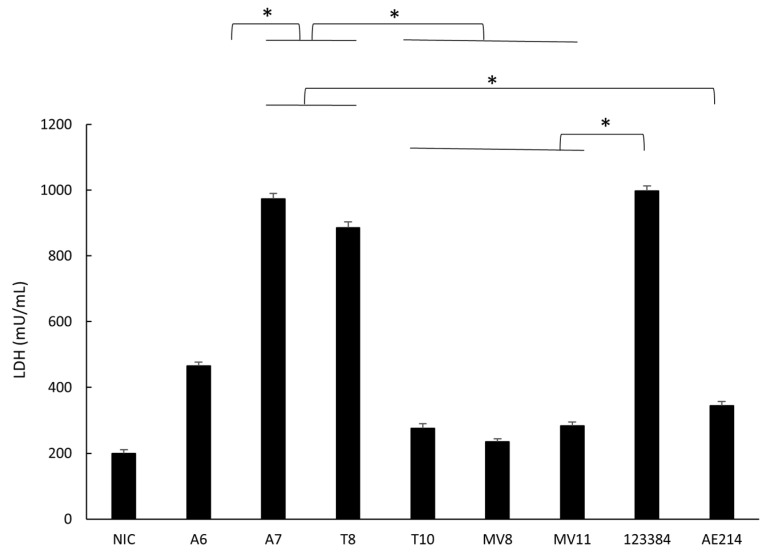
Determination of the cell damage to macrophages (J744A.1) induced by the six selected *Aeromonas* strains (A6 and A7 from water; T8 and T10 from soil; and MV8 and MV11 from vegetation) and by *Aeromonas veronii* 123384 and *Aeromonas jandaei* AE214, at MOI 10 after 3 h of incubation. Cell damage was evaluated measuring the release of lactate dehydrogenase (LDH). * Significant differences compared with non-infected cells (NIC) *p* < 0.05. Results are means ± SD from at least three independent experiments.

The six strains caused a significantly higher level of cell damage (*p* < 0.05) after infection of the macrophages at MOI 10 compared with the non-infected cells, as measured by the LDH released. The strains A7 (*Aeromonas* sp.) and T8 (*A. popoffii*) caused a higher level of cell damage than the other strains, similar to the one caused by the control *A. veronii* 123384 (Figure 5).

### 3.8. Intracellular Bacterial Survival of Aeromonas Strains

**Figure 6:**
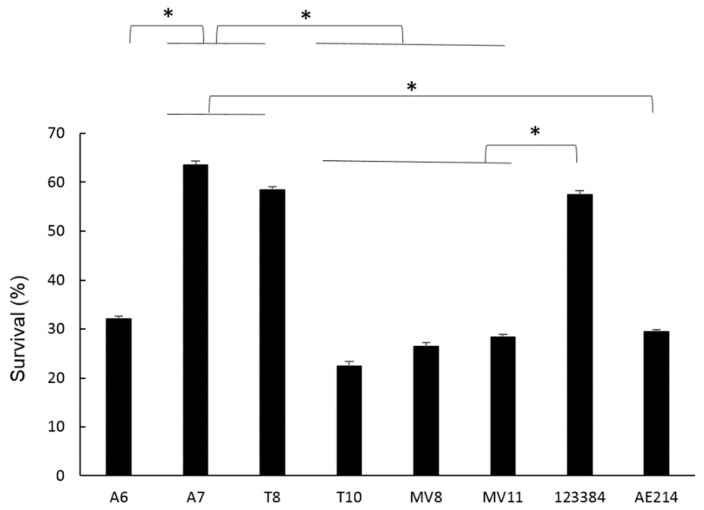
Percentages of intracellular survival of six *Aeromonas* strains (A6 and A7 from water; T8 and T10 from soil; and MV8 and MV11 from vegetation) and of *Aeromonas veronii* 123384 and *Aeromonas jandaei* AE214, in macrophages (J744A.1) at MOI 10 after 3 h of incubation. Percentages were calculated with respect to non-infected cells. * Significant differences compared with non-infected cells *p* < 0.05. Results are means ± SD from at least three independent experiments.

The percentage of intracellular survival of the six *Aeromonas* strains at MOI 10 after 3 h of incubation was calculated after serial dilution and plating and it is shown in Figure 6. Significant differences were observed when comparing the survival of A7 (*Aeromonas* sp.) and T8 (*A. popoffii*) strains to the rest, with survival rates of 60% and 50% (*p* < 0.05), respectively. With the rest of the strains, no significant differences were observed between them, with intracellular survival at 3 h being approximately between 20–30%.

### 3.9. Antimicrobial Susceptibility Profile

The results of the antimicrobial susceptibility test are showed in Table 3. All strains were categorized as resistant to ampicillin, and none of them showed in vitro resistance to tetracycline. For the rest of the antibiotics tested, the percentage of resistant strains was variable: 50% for cefuroxime and 33.3% for both ceftriaxone and piperacillin/tazobactam.

## 4. Discussion

Our work focused on the presence of *Aeromonas* in the natural area “El Clot de la Mare de Déu”, which belongs to the fluvial area of the Mijares river. The waters of this area are supplied by an underground spring and flow into the Mediterranean Sea. These waters are used for human recreation, especially in the warmer months of the year, and to irrigate the adjacent crop fields without being subjected to any type of depuration treatment. The aim was to determine the incidence of *Aeromonas* in this recreational environment, the potential virulence of the strains isolated, and their antimicrobial susceptibility, considering the fact that cases of wound infections can evolve as severe cases of necrotizing fasciitis [17,18,46,47], which is a life-threatening infection if proper antibiotic treatment is delayed. On this basis, some authors consider *Aeromonas* a flesh-eating bacteria and an emerging aggressive pathogen [17,18,48]. An impacting case occurred in a healthy immunocompetent woman, who fell into a river while practicing a zip line and an open wound got contaminated with *Aeromonas*, generating a fast-evolving necrotizing fasciitis that required progressive amputations of large parts of her limbs to survive [17,18]. 

In our study, the presence of *Aeromonas* was confirmed at high concentrations in samples of water, soil, and vegetation with the techniques used (qPCR, MPN, and Plate Counting in ADA medium). In general, our results showed that there was a relatively high concentration of *Aeromonas* in all of the samples, in line with the concentration normally found in rivers, lakes, and other natural reservoirs (up to 3.4 × 10^4^ and 6.9 × 10^3^ CFU/100 mL) [9], as well as in waters used for irrigation (from 7.0 × 10^2^ CFU/100 mL to 2.45 × 10^4^ CFU/100 mL) [8]. 

As expected, the technique that showed a higher concentration of *Aeromonas* compared to the other techniques was the qPCR, due to the high degree of sensitivity and because it is not able to discriminate between live and dead bacteria [49,50]. The quantification technique that showed a lower concentration of *Aeromonas* was MPN, because it provides an approximate result. In the case of plate counting in ADA medium, it was observed that there was an intermediate value of concentration between the values obtained with qPCR and with the MPN. It has been observed that in ADA medium, other bacteria are able to grow, as proven in previous studies [51]. For this reason, there is a need to perform the three methods for a more reliable *Aeromonas* quantification.

To study the diversity of *Aeromonas* spp. in the samples, 24 colonies that showed the typical morphology described for *Aeromonas* grown in the ADA culture media plates were selected. Of the selected 24 isolates, only 19 (79%) belonged presumptively to *Aeromonas* on the basis of the presence of the GCAT gene, which is considered specific for the genus [29,52], indicating that the ADA generated, in this case, 21% of false positives.

The phylogenetic analysis constructed with the sequences of the *rpoD* gene of the 19 isolates identified them as belonging to seven known *Aeromonas* species and one as a potential new species. These results corroborate that the GCAT and the *rpoD* genes are excellent tools for the identification of the members of the genus *Aeromonas* and for recognizing known and potentially new species [12,29,31,52]. The latter seems to be the case for strain A7, isolated from water, which clustered in the phylogenetic tree at a significant distance from all the *Aeromonas* species known so far. Further studies that sequence either the genome of this strain or additional housekeeping genes would be necessary to confirm this finding [53]. It was also observed that two of the strains isolated from vegetation clustered with the species *A. media* and *A. rivipollensis,* but phylogenetically are close to the not-yet-described genomic candidate species “*A. paramedia*”. The clonal relationships between strains was confirmed on the basis of their identical *rpoD* sequences and the identical ERIC-PCR patterns, demonstrating that water and soil samples are colonized by the same bacteria. Previous studies showed that water acts as a vector for the transmission of *Aeromonas* to other substrates [8], as our results also corroborate. 

The pathogenic potential of six *Aeromonas* strains was evaluated by the presence of five virulence genes related to the flagellum mobility (*laf*), the Type III secretion system (*ascF-G*), and toxins (*alt*, *ast* and *stx1*). There was a great variability between strains, as shown in previous studies [12,22]. All the strains were positive for the presence of the *laf* gene, encoding for structural protein of the lateral flagellum [32]. The presence of lateral flagellum gives the bacteria a fast or “swarming” type of mobility, which allows them to move on solid surfaces and form biofilms expressed during bacterial growth on viscous surfaces [22,23,32,54,55,56]. The presence of *alt* and *ast* genes, encoding for thermolabile and thermostable cytotonic enterotoxins, and the expression level of *ast* were variable between strains. The cytotonic enterotoxins have a similar mechanisms of action of the choleric toxin, increasing the cyclic adenosine monophosphate (cAMP) levels and prostaglandins in the intestinal epithelial cells [22]. On the other hand, the *stx1* gene, encoding for a Shiga-like toxin [57], was not present in any of the strains studied. Previous reports have detected Shiga-like toxins in clinical strains of *Aeromonas* [58] and some strains recovered from food [12]. The Shiga toxin inactivates ribosomes (arrest of protein synthesis) of vascular endothelial cells, leading to cell death [57]. Finally, the presence of the *ascF*-G gene, which encodes for Type III Secretion System (T3SS) [36], was detected in four of the strains and the expression level was different between the strains. The T3SS has been previously observed as having a great role in the virulence of *Aeromonas* [36,59,60]. This is one of the secretion systems by which proteins can be injected directly from the bacterial cell protoplasm to the cytoplasm of the target cell or to the extracellular space [61,62]. 

In the in vitro infection assay, two parameters were studied—the cell damage caused in the macrophage cell line J774A.1 and the intracellular survival of the bacteria within the macrophages. These parameters were used as indicators of the ability of *Aeromonas* strains to induce an infection. The measuring of cell damage in animal cell models through quantification of the enzyme LDH, released during apoptosis or pyroptosis, is a well-established method [63]. It was observed that the strains A7 from river water and T8 from soil caused a greater cell damage in the macrophages than the other strains. Some *Aeromonas* infection studies have used this method to assess the pathogenicity of the strains tested, like Epple et al. [64], in which colon epithelial cells HT-29/B6 were infected with *A. hydrophila* and *A. veronii* strains isolated from stool. Regarding the obtained percentages of intracellular bacterial survival, these were higher for the strains A7 and T8 compared to the other strains after 3 h of infection. Studies with *Aeromonas* environmental strains have shown that these are also capable, like clinical strains, of invading and surviving within different cell lines. For example, Couto et al. [65] demonstrated that *A. hydrophila* and *A. caviae* strains isolated from human diarrheic feces, vegetables, and water were able to adhere and invade different intestinal epithelial cell lines and produce cytotoxic and cytopathic effects. In another study, dos Santos et al. [66] demonstrated that eight *Aeromonas* spp. strains isolated from human feces, food, and water were able to invade intestinal (T-84, Caco-2) and epithelial (HEp-2) cell lines cultivated in vitro. Dias et al. [67] proved that *A. salmonicida* isolated from wild animal feces exhibited the highest ability to internalize and survive in Caco-2 cells under simulated human gastrointestinal conditions among other bacteria tested. 

*Aeromonas*, like other Gram-negative bacilli, are intrinsically resistant to benzylpenicillin, glycopeptides, lipoglycopeptides, fusidic acid, macrolides, lincosamides, streptogramins, rifampicin, and oxazolidinones, such that, these drugs should not be considered for either therapy or clinical susceptibility testing [68]. Previous literature has shown that these bacteria present a high level of resistance to antibiotics, such as aminopenicillins and their beta-lactamase inhibitor combinations, and first-generation cephalosporins, with few exceptions [12,68,69]. In contrast, antimicrobial agents, such as third- and fourth-generation cephalosporins, carbapenems, monobactams, piperacillin-tazobactam, aminoglycosides, fluoroquinolones, and cotrimoxazole show in vitro activity against these bacteria [11,12,70,71]. However, the resistance of *Aeromonas* to these drugs has increased in recent decades, both in clinical and environmental isolates [12,71,72]. The use of these antibiotics for prophylactic and therapeutic purposes, both in humans and animals, mainly in fish, has probably influenced the increase in this acquired resistance. 

In our work, antimicrobial susceptibility profiles of six *Aeromonas* strains against five antimicrobial agents (ampicillin, cefuroxime, ceftriaxone, tetracycline, and piperacillin/tazobactam), which are frequently used as empirical treatments for a wide range of bacterial infections, were analyzed. In this assay, as could be expected, all strains were resistant to ampicillin. Our results are in accordance with previous published studies describing *Aeromonas trota* as the only species of the genus susceptible to this aminopenicillin [73,74,75,76,77,78]. Variable resistances to other beta-lactam antibiotics tested, including piperacillin/tazobactam, cefuroxime, and ceftriaxone, were observed. The resistance to these drugs of some strains was probably due to the production of beta-lactamase enzymes, such as extended-spectrum beta-lactamases (ESBLs), metallo-beta-lactamases, cephalosporinases, and penicillinases, which confer resistance to most beta-lactam antibiotics, including penicillin, cephalosporins, carbapenems, and the monobactam aztreonam [11,79,80,81]. In the study of Piotrowska et al. [82], it was observed that the different genes encoding beta-lactamases present in *Aeromonas* species isolated from sewage were mainly found in plasmids. This suggested that *Aeromonas* resistance to this group of antimicrobials could spread from water residues to other substrates, thus increasing the biosanitary risk involved considering that third- and fourth-generation cephalosporin regimes have been used to treat systemic infections in humans [69]. Finally, in regard to tetracycline, we did not detect any strain resistant to this drug. The main mechanisms of bacterial resistance to the tetracycline group are the ribosomal protection and the efflux pump, both generally associated with the presence of *tet* genes. The *tet* genes encode different cytoplasmic proteins (Tet) that can interact with ribosomes, preventing tetracycline from binding to its target, and can interact with tetracycline, behaving as active exporters of the drug to out of the cell [83]. In general, higher percentages of tetracycline resistance have been described in clinical strains than in environmental strains, although an increase in such resistance has also been detected in the latter [84,85].

## 5. Conclusions

This work constitutes a preliminary study that will continue with a more exhaustive investigation to determine the risk assessment associated with the presence of pathogenic *Aeromonas* spp. in these waters. With the techniques used here, high concentrations of *Aeromonas* spp. were found in water, soil, and vegetation samples of this natural recreational environment. Considering the pathogenic potential of some of the isolated strains, the presence of this bacteria may represent a threat in the case of exposed open wounds. Furthermore, the antimicrobial susceptibility profile of the strains studied confirm the increased resistance to antibiotics of these bacteria, endangering the ability to treat infections. The presence of clones among the different samples supports the hypothesis that water can act as a transmission vector of *Aeromonas* to other substrates. This study confirmed once more that the use of the sequences of the *rpoD* enables the identification and recognition of known and potential new species of *Aeromonas*. 

## Figures and Tables

**Table 1:** Primers used in this study.

Primer	Sequence 5′-3′	Target	Reference
GCAT-F	CTCCTGGAATCCCAAGTATCAG	*GCAT*	[29]
GCAT-R	GGCAGGTTGAACAGCAGTATCT		
ERIC 1R	ATGTAAGCTCCTGGGGATTCAC	Genome	[30]
ERIC 2	AAGTAAGTGACTGGGGTGAGCG		
RpoD-Fs	GTCAATTCCGCCTGATGC	*rpoD*	[31]
RpoD-Rs	ATCATCTCGCGCATGTTGT		
Laf1	GGTCTGCGCATCCAACTC	*lafA*	[32]
Laf2	GCTCCAGACGGTTGATG		
alt-F	AAAGCGTCTGACAGCGAAGT	*alt*	[33]
alt-R	AGCGCATAGGCGTTCTCTT		
ast-F	ATCGTCAGCGACAGCTCTT	*ast*	[34]
ast-R	CTCATCCCTTGGCTTGTTGT		
Stx1-a	TCTCAGTGGGCGTTCTTATG	*stx1*	[35]
Stx1-b	TACCCCCTCAACTGCTAATA		
ascF-G-F	ATGAGGTCATCTGCTCGCGC	*ascF-G*	[36]
ascF-G-R	GGAGCACAACCATGGCTGAT		

**Table 2:** Presence of virulence genes in 6 *Aeromonas* strains isolated from the natural area “El Clot de la Mare de Dèu”.

Gene	Product	Reference	A6	A7	T8	T10	MV8	MV11
*laf*	Lateral flagella structural protein	[32]	+	+	+	+	+	+
*alt*	Cytotonic enterotoxin	[33]	+	+	+	-	-	+
*ast*	Cytotonic enterotoxin	[34]	+	+	+	+	-	+
*stx*1	Shiga toxin	[35]	-	-	-	-	-	-
*ascF-G*	T3SS structural protein	[36]	-	+	+	-	+	+

**Table 3:** Antimicrobial susceptibility profile of six *Aeromonas* strains from water (A6, A7) soil (T8, T10) and vegetation (MV8, MV11) isolated from the natural area “El Clot de la Mare de Dèu”.

Antimicrobial (µg)	A6	A7	T8	T10	MV8	MV11
Ampicillin (10)	R	R	R	R	R	R
Cefuroxime (30)	I	S	I	R	R	R
Ceftriaxone (30)	S	S	S	I	R	R
Tetracycline (30)	S	I	I	I	I	I
Piperacillin/tazobactam (100/10)	S	S	R	I	I	R
